# Covariate balance in a Bayesian propensity score analysis of beta blocker therapy in heart failure patients

**DOI:** 10.1186/1742-5573-6-5

**Published:** 2009-09-10

**Authors:** Lawrence C McCandless, Paul Gustafson, Peter C Austin, Adrian R Levy

**Affiliations:** 1 Faculty of Health Sciences, Simon Fraser University, Canada; 2 Department of Statistics, University of British Columbia, Canada; 3 Institute for Clinical Evaluative Sciences, Toronto, Canada; 4 Dalla Lana School of Public Health, University of Toronto, Canada; 5 Department of Health Policy, Management and Evaluation, University of Toronto, Canada; 6 School of Population and Public Health, University of British Columbia, Canada

## Abstract

Regression adjustment for the propensity score is a statistical method that reduces confounding from measured variables in observational data. A Bayesian propensity score analysis extends this idea by using simultaneous estimation of the propensity scores and the treatment effect. In this article, we conduct an empirical investigation of the performance of Bayesian propensity scores in the context of an observational study of the effectiveness of beta-blocker therapy in heart failure patients. We study the balancing properties of the estimated propensity scores. Traditional Frequentist propensity scores focus attention on balancing covariates that are strongly associated with treatment. In contrast, we demonstrate that Bayesian propensity scores can be used to balance the association between covariates and the outcome. This balancing property has the effect of reducing confounding bias because it reduces the degree to which covariates are outcome risk factors.

## Introduction

Regression adjustment for the propensity score is a statistical method that reduces confounding from measured variables in observational data. The idea is to use the propensity score, defined as the probability of treatment given measured confounders, to build treatments groups that are similar with respect to outcome risk factors [1]. Patients with the same propensity score have the same distribution of measured confounders. Provided that there is no unmeasured confounding, we obtain unbiased estimates of the treatment effect by comparing treatment groups within levels of the propensity score. Analytic techniques using propensity scores include stratifying on quintiles of the propensity score, or using the propensity score as a covariate in a regression model for the outcome. Other methods using matching or weighting are available [2].

Recently, McCandless, Gustafson and Austin [3] proposed a statistical method which combines regression adjustment for the propensity score with Bayesian techniques. Their proposed Bayesian propensity score analysis (BPSA) models the propensity score as a latent variable that is integrated from the posterior distribution for the treatment effect. BPSA fits regression models for the outcome and treatment simultaneously rather than one at a time. When estimating the propensity scores, BPSA incorporates prior information about the relationship between the outcome and propensity score within treatment groups. In contrast, standard analytic methods estimate propensity scores from the marginal model for treatment given measured confounders.

Other Bayesian techniques using propensity scores are given by Hill and McCulloch [4] and Hoshino [5]. The approach of Hill and McCulloch [4] uses nonparametric modelling of the outcome using Bayesian additive regression trees. It has the advantage that the user is not required to supply modeling assumptions about the manner in which variables are parametrically related. Hoshino describes a Markov chain Monte Carlo technique for fitting propensity models to observational data [5].

McCandless et al. [3] use simulations to evaluate the performance of BPSA. They demonstrate that if the regression model for the relationship between outcome and propensity score is correctly specified, then BPSA permits more efficient estimation of the propensity scores compared to other non-Bayesian methods. However, when the outcome regression model is incorrectly specified, this can adversely impact BPSA and give propensity score estimates that are asymptotically biased. Thus it is unclear whether BPSA will outperform standard non-Bayesian approaches in real data applications. In practice, statistical models for the outcome variable are only approximations.

An appealing feature of propensity score techniques is that simple diagnostics tools have been developed to compare the performance of competing propensity score estimates for control of confounding. Conditional on the propensity score, treatment and confounders are independent. The distribution of the confounders should be similar across treatment groups. This can be empirically verified by comparing summary statistics such as the mean and variance for covariates in treatment versus control. If the distributions are similar then this indicates that treatment effect estimates are unconfounded. Following convention in the literature, we refer to this diagnostic procedure as checking covariate *balance*. A detailed discussion is given by Austin and Mamdani [2].

The balancing properties of propensity score estimates have been well studied. Austin and Mamdani [2] and Austin et al. [6] studied the impact of different variable selection strategies on the balancing properties of estimated propensity scores. The authors show that including non-confounders in the model for the propensity score can reduce the amount of covariate balance on the confounders. Matching on the propensity score produces greater balance compared to stratifying on quintiles of the propensity score. Austin et al. [7] investigated covariate balance in settings where there are unmeasured confounders.

The logic of checking covariate balance provides an opportunity to evaluate the performance of BPSA. We can empirically verify if the propensity score estimates yield similar balance compared to conventional methods. Accordingly, our objective is to study covariate balance for BPSA. In what follows, we present a case-study of an observational study of the effectiveness of beta-blocker therapy in British Columbia heart failure patients. In the example, there is strong confounding because beta-blockers are preferentially prescribed to younger, healthier patients. We analyze the data using BPSA and compute propensity estimates from the posterior distribution of the propensity scores. We study balance with respect to treatment when stratifying on the estimated propensity scores. Our analysis reveals that BPSA gives worse balance compared to conventional propensity scores estimates calculated from the marginal model for treatment. The covariate distributions differ in treatment versus control. However, we then show that BPSA yields improved in balance with respect to the outcome. By this we mean that stratifying on BPSA propensity score estimates reduces the strength of the association between the covariates and mortality. This reduces confounding bias because it reduces the impact of covariate imbalances between treatment groups. Thus BPSA makes a tradeoff between balancing baseline covariates with respect to the treatment versus the outcome variable. In contrast, conventional propensity score estimates are calculated from the marginal model for treatment. They handle all confounders equally, regardless of whether they are important outcome risk factors.

## Estimating the effectiveness of beta-blocker therapy in heart failure patients

To investigate the ability of BPSA to control confounding, we consider the example of an observational study of the effect of beta-blockers on one year all-cause mortality in heart failure patients from British Columbia. Beta-blockers are a class of cardiovascular therapies which act on the beta-adrenergic nervous system to improve heart function [8]. Randomized trials show that they reduce in mortality in heart failure patients, but there is interest in quantifying the magnitude of this effect within the general population, including the very elderly [9]. In Canada, beta-blockers are more often prescribed to patients who are young, healthy and with fewer comorbidities [10]. Because treated patients in the population are healthier than untreated patients, we expect that a crude comparison of mortality rates will be confounded and tend to exaggerate the benefits of beta-blocker therapy. Treated patients will have lower mortality because they are in better health, even in the absence of any benefit of beta-blockers.

In this study, we obtained administrative health data for one year of follow-up on 6969 patients discharged from British Columbia hospitals in 1999 and 2000. Using records of hospitalization and drug prescription claims, we compiled information on demographic characteristics, comorbid medical conditions and medications dispensed from community pharmacies throughout the province. Vital status at the end of follow up was established by electronic linkage of medical records to death certificates. Full details are provided elsewhere [8,11]. After one year, 1755 patients died, and the mortality rate among treated patients was 19% versus 27% among untreated patients. The crude odds ratio for the association between beta-blockers and mortality is 0.64 with 95% credible interval (0.55, 0.75). In contrast, meta-analyses of randomized controlled trials consistently report a 30% reduction in mortality with beta blocker use [9]. This suggests that the association between beta-blocker therapy and mortality is confounded due to measured and unmeasured indications for disease severity. To estimate the treatment effect analytic adjustments are required, and this provides a test case for comparing the performance of BPSA with other methods.

### Bayesian propensity score analysis: Data, models and estimation

Let *X *denote a binary variable representing exposure to beta-blockers. We set *X *equal to one if the subject was dispensed a beta-blocker within 30 days of discharge from hospital and zero otherwise. The binary response variable *Y *is set equal to one if the subject died within one year of discharge from hospital and zero otherwise. Let *C *= (*C*_1_, *C*_2_,..., *C*_*p*_) denote a vector of *p *= 21 potential confounding variables measured on or before hospital discharge including demographic characteristics: age (categorical with four levels; <65, 65-74, 75-84, ≥ 85 years), sex (binary with one indicating female and zero otherwise); indicator variables for comorbid conditions: cerebrovascular disease, chronic obstructive pulmonary disorder (COPD), hyponatremia, metastatic disorder, renal disease, ventricular arrhythmia, liver disease, malignancy, shock; indicator variables for dispensation of heart failure medications within thirty days of hospital discharge: angiosin converting enzyme (ACE) inhibitors, angiotensin II receptor blockers (ARB), calcium channel blockers (CCB), digoxin, diuretics, statins; and characteristics of the index hospitalization: indicator of transferred status, hospital length of stay in days. In order to ease the specification of intercept terms in regression modelling, we set the first component of *C *(denoted *C*_0_) to be equal to one.

To model the propensity score and relationship between *Y*, *X *and *C*, we use two logistic regression models. Following McCandless et al. [3], we let

(1)

(2)

The quantity *β *models the treatment effect, while the parameter *γ *= (*γ*_0_,..., *γ*_*p*_) is a (*p *+ 1) × 1 vector of regression coefficients which identifies the propensity score, given by *Z *= logit{*Pr*(*X *= 1|*C*)} = *γ*^*T*^*C*. Following Rubin and Thomas [12], we define the propensity score as the log odds of treatment given measured confounders. This definition differs slightly from the usual definition in the literature, but it eases the analytical tractability of studying propensity score estimates. In practice, both definitions give similar treatment effect estimates because the log odds transformation is monotonic [12].

In equation (1), we use regression splines to flexibly model the nonparametric relationship between the propensity score and outcome variable. In the summation , the quantities *g*_*j *_{·}, *j *= 1,..., *l *= 3 are natural cubic spline basis functions with *l *= 3 knots (*q*_1_, *q*_2_, *q*_3_), and regression coefficients *ξ *= (*ξ*_1_, *ξ*_2_, *ξ*_3_). The choice of *l *= 3 knots reflects a trade off between smoothness and complexity. Alternatively, we could use hierarchical models to model uncertainty in the location or number of knots.

We assign prior distributions for the model parameters *β*, *γ*, *ξ *as



where  =  =  = {log(15)/2}^2^. The value for  models the belief that the odds ratio for the treatment effect is not overly large and lies between 1/15 and 15 with probability 95%. The values for  and  make similar modelling assumptions about the prior magnitude for the association between *Y *and *Z *given *X*, and also the association between *C *and *X*.

The regression models in equations (1) and (2) give a likelihood function for the data. Combining the likelihood and prior distributions, we can sample from the posterior distribution of the treatment effect *β *and nuisance parameters *γ*, *ξ *using Markov chain Monte Carlo. Conceptionally, the implementation involves a two step iterative procedure: First, impute the propensity score parameter *γ*. Second, fit a complete data step to estimate the treatment effect *β *and *ξ *given the propensity scores. Successive iterations average over uncertainty in the propensity scores. The approach has close connections to the EM algorithm and multiple imputation. Computer code for implementing BPSA in the software package R [13] is available [see additional file 1]. A detailed discussion of implementing BPSA is given in McCandless et al. [3].

Before applying BPSA to the data, we first select the knots used in the spline regression for the relationship between mortality and propensity score. To choose the knots, we fit the logistic regression model given in equation (2) via maximum likelihood and compute the fitted values. The three knots are chosen as *q*_1 _= 0.10, *q*_2 _= 0.18, *q*_3 _= 0.24, which define quartiles of the estimated propensity scores. We then apply BPSA to the data by sampling from the posterior density *P*(*β*, *ξ*, *γ*|*data*). We run a single MCMC chain of length 100 000 after discarding 10 000 initial iterations. Sampler convergence is assessed by simulating separate MCMC chains with overdispersed starting values and the diagnostic tools supplied in the CODA package in R [13].

### Analysis results

The results are given in Table 1 under the heading "BPSA", which contain posterior means and 95% credible intervals for the treatment effect *β *and the regression coefficients *γ*. We omit estimates of *ξ *because the quantity is a nuisance parameter with an interpretation that depends on the parameterization of the natural splines in equation (1).

**Table 1:** Log odds ratios (95% CIs) for the treatment effect *β *and the regression coefficients *γ *calculated using BPSA and PSA.

**Description**	**Parameter**	**Log Odds Ratio (95% Interval Estimate)**	
		**BPSA**	**PSA**
Beta blocker	*β*	-0.21 (-0.37, -0.05)	-0.31 (-0.46, -0.15)
*Demographics*			
Female Sex	*γ*_1_	0.17 (0.06, 0.29)	0.12 (-0.01, 0.25)
Age			
< 65 (reference)	.	0.00	0.00
65 - 74	*γ*_2_	-0.19 (-0.32, -0.04)	-0.09 (-0.3, 0.12)
75 - 84	*γ*_3_	-0.40 (-0.56, -0.24)	-0.21 (-0.41, 0.00)
> 85	*γ*_4_	-0.71 (-0.94, -0.46)	-0.37 (-0.59, -0.14)
*Comorbid conditions*			
Cerebrovascular dis.	*γ*_5_	-0.11 (-0.67, 0.44)	0.25 (-0.46, 0.96)
COPD	*γ*_6_	-0.32 (-0.60, -0.06)	-0.89 (-1.30, -0.49)
Hyponatremia	*γ*_7_	-0.02 (-0.26, 0.21)	0.03 (-0.33, 0.39)
Metastatic disorder	*γ*_8_	-1.42 (-2.33, -0.56)	-0.40 (-1.37, 0.57)
Renal disease	*γ*_9_	-0.17 (-0.32, 0.01)	0.38 (0.15, 0.62)
Ventricular arrhythmia	*γ*_10_	-0.12 (-0.63, 0.44)	0.12 (-0.62, 0.86)
Liver disease	*γ*_11_	-0.52 (-1.03, -0.08)	-1.11 (-2.04, -0.19)
Malignancy	*γ*_12_	-0.78 (-1.19, -0.34)	-0.06 (-0.57, 0.45)
Shock	*γ*_13_	-0.06 (-0.56, 0.39)	-0.12 (-0.83, 0.58)
*Hospitalization*			
Transferred	*γ*_14_	-0.41 (-0.58, -0.25)	-0.01 (-0.22, 0.20)
Stay (10 day intvs.)	*γ*_15_	-0.13 (-0.18, -0.09)	-0.05 (-0.11, 0.01)
*Heart failure medications*			
Digoxin	*γ*_16_	-0.02 (-0.11, 0.07)	0.00 (-0.14, 0.13)
Diuretic	*γ*_17_	0.28 (0.09, 0.48)	0.72 (0.54, 0.90)
CCB	*γ*_18_	0.22 (0.08, 0.35)	0.27 (0.10, 0.44)
ACE inhibitor	*γ*_19_	0.29 (0.11, 0.45)	0.61 (0.47, 0.76)
ARB	*γ*_20_	0.18 (-0.06, 0.46)	0.53 (0.19, 0.87)
Statin	*γ*_21_	0.91 (0.65, 1.24)	0.94 (0.76, 1.12)

While the priors distributions are plausible, they may nonetheless be informative. We repeat the analysis by fixing the prior variances equal to 10^3 ^rather than {log(15)/2}^2^. Additionally, we experiment with uniform priors bounded on the interval [-10, 10]. The resulting inferences for *β *are similar to those in Table 1 and differ by less than 0.03 on the log odds scale. For the parameter *γ*, the MCMC output is similar under different priors, although there is less shrinkage towards origin using the uninformative priors. Posterior means differed by at most 0.05 for all covariates except metastatic disorder and malignancy.

For comparison, we also apply a propensity score analysis (PSA) to the data. We define PSA as the following two step procedure: First, fit the logistic regression model in equation (2) by maximum likelihood and compute the estimated propensity scores from the fitted values. Next, fit the model in equation (1) by maximum likelihood, substituting the fitted values in place for the true propensity scores. PSA is a standard method for controlling confounding [2]. It is identical to BPSA, except that it fits the regressions models in equations (1) and (2) one at at time rather than simultaneously. PSA is implemented using the same knots (*q*_1_, *q*_2_, *q*_3_) as BPSA. The results are given in the second column of Table 1 under the heading "PSA".

As expected, the estimates for the treatment effect *β *from BPSA and PSA are less than zero and the interval estimates exclude zero, indicating that beta-blockers reduce mortality in heart failure patients. But the treatment effect estimates are slightly different. BPSA gives an odds ratio of exp(-0.21) = 0.81 whereas PSA gives exp(-0.31) = 0.73. BPSA and PSA also give different inferences for *γ*. The differences in point estimates of *γ *are substantial, although they are generally small compared to the 95% interval estimates. From equation (2), we can see that the parameter *γ *models the propensity score in the sense that if *γ *is known then the propensity score for a patient with covariate vector *C *is given by *γ*^*T*^*C*. Because the estimates of *γ *differ for BPSA versus PSA, this suggests that the propensity score estimates also differ.

Which inferences should we prefer? BPSA and PSA use identical models, but yield qualitatively different answers. The reason is because BPSA fits regression models for *Y *and *X *simultaneously rather than one at at time. McCandless et al. [3] conducted detailed simulations and showed that if the outcome model in equation (1) is sufficiently non-parametric to capture the dependence between *Y *and *Z*, then BPSA gives more efficient estimates of the propensity scores. But for the heart failure data the true data generating process is unknown.

**Figure 1:**
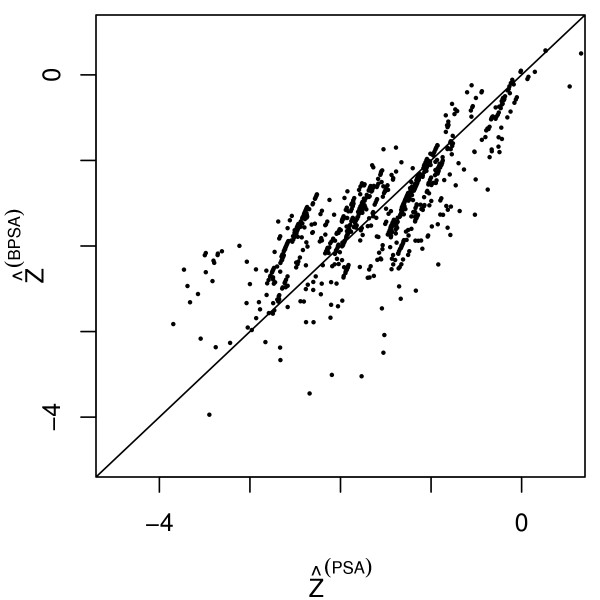
** versus  for a random sample of 1000 subjects in the heart failure study**.

To explore the results in greater detail, we compare the estimated propensity scores from either method. Let  denote the estimate for *γ *obtained from PSA and let  denote the posterior mean for *γ *from BPSA. The estimated propensity score from PSA is , whereas for BPSA it is . Figure 1 plots  versus  for a random sample of 1000 subjects in the study. The quantities have correlation equal to 0.85, but their dependence is nonetheless heterogeneous. The linear clustering in the figure is due to the covariate for hospital length of stay. This is the only continuous covariate in the dataset, with median length of stay equal to 5 days and interquartile range of 3 to 10 days. We see in Table 1 that BPSA and PSA give different estimates for *γ*_15 _which models the relationship between length of stay and treatment assignment. The clusters in Figure 1 are groups of patients who spent different amounts of time in hospital, but otherwise have the same covariate pattern.

In PSA we control confounding by stratifying on , whereas in BPSA we stratify on . Figure 1 shows that the methods stratify subjects into different groups. Provided that the models in equations (1) and (2) are correct, then large sample Bayesian theory tells us that the parameter estimates for *γ *calculated from BPSA and PSA will be asymptotically identical and consistent to the true parameter value [14]. But for the heart failure data, the combination of a finite sample size and possible model misspecification leads to sizeable differences in the propensity score estimates.

One of the attractive properties of propensity score techniques is that simple diagnostic tools are available to study the performance of competing propensity score estimates. If we condition on the propensity score, then the treatment and confounders are independent. The empirical distribution of the confounders should be balanced across treatment groups, and this can be verified by comparing the mean and variance of covariates in treatment versus control. If the distributions are similar then this indicates that the confounding has been reduced [12]. We use the notion of covariate balance as a starting point for evaluating BPSA versus PSA.

## Balance with respect to treatment

In this section, we investigate the balancing properties of  and . Rosenbaum and Rubin [1] showed that *X *╨*C*|*Z*, where the symbol "╨" means that *X *and *C *are conditionally independent given the true propensity score *Z*. Stratifying on the propensity score confers *balance with respect to treatment *and breaks the association between *X *and *C*. To investigate the performance of competing propensity score estimates, we adopt an approach similar to Imai and Van Dyk [15] and fit models of the form

(3)

where *C*_*k *_denotes the *k*^*th *^component of *C*. Equation (3) is identical to equation (1) except that it substitutes each of the covariates in place of the outcome variable *Y*. To understand the logic behind fitting such a model to assess balance, notice that if  is equal to the true propensity score, then this implies that *θ*_1 _= *θ*_2 _= *θ*_3_= ... = *θ*_21 _= 0 in equation (3) because *X *╨ *C*_*k*_| for each of *k *= 1,..., 21. Thus the extent to which estimates of *θ*_1_,..., *θ*_21 _depart from zero speaks to the balancing properties of competing propensity score estimates, and therefore, the effectiveness of BPSA and PSA in control of confounding. If *X *╨ *C*_*k*_|, then 95% interval estimates for *θ*_*k *_should cover zero with probability 95%.

This reasoning is analogous to investigating balance by reporting covariate summary statistics within quintiles of the estimated propensity scores. The original approach of assessing balance recommended by Rosenbaum and Rubin [1] proceeds as follows: First, estimate the propensity score via regression of treatment on covariates. Next, break the population into five separate quintile groups based the estimated propensity scores. Lastly, check balance within each quintile group by comparing the distribution of covariates (e.g. age) in treated versus untreated. If the distributions are similar, then this suggests that the estimated propensity scores succeed in breaking the association between treatment and confounders. Equation (3) assesses balance in a similar fashion. We regress *C*_*k *_on *X *and . If the regression coefficient *θ*_*k *_is zero, then this means that *X *and *C*_*k *_are not associated after having stratified on . Therefore  is a "good" propensity score estimate because it induces balance with respect to treatment.

**Figure 2:**
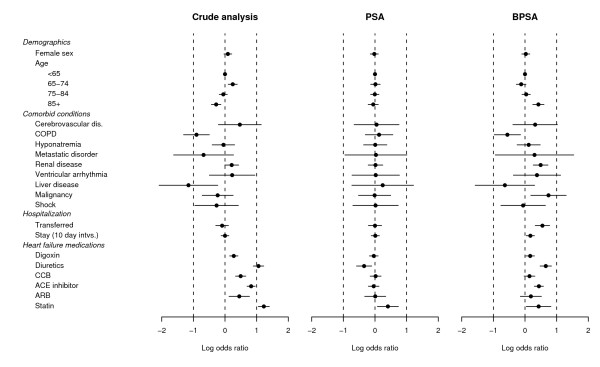
**Balance with respect to treatment**. Each row corresponds to the log odds ratio (95% CI) for the association between a covariate and treatment in either an unadjusted analysis, or after having adjusted for  or .

To illustrate in the heart failure data, we begin by studying the crude associations between *X *and *C*. This is accomplished by fitting the 21 regressions in equation (3) while forcing *ω*_*jk *_equal to zero. In other words, we individually regress the components of *C *on *X*. The variable for hospital length of stay, which is continuous, it dichotomized at the sample median. The results, in the form of point and 95% interval estimates of *θ*_1_,..., *θ*_21 _are plotted in the first column of Figure 2 under the heading "Crude analysis". For example, for *C*_1 _which indicates female sex, we estimate *θ*_1 _as 0.09 with 95% confidence interval (-0.01, 0.18), and this result is plotted accordingly. Figure 2 reveals that many covariates are associated with of treatment. In particular, treated patients are more likely to be treated with other heart failure medications.

Next we examine the performance of  as a tool to reduce confounding. We fit the 21 regressions in equation (3) by substituting  and compute point and interval estimates for *θ*_1_,..., *θ*_21_. The results are given in the second column of Figure 2 under the heading "PSA". Here we see the that the estimates  are close to zero because the same data are used to estimate the propensity scores and to check balance with respect to *X*. This illustrates the ability of PSA to control confounding. Compared to the crude analysis, Figure 2 reveals that stratifying on  breaks the association between *C *and *X*, and thus reduces confounding.

Finally, we repeat the above regressions substituting  in place for  in equation (3). We calculate point and interval estimates for *θ*_1_,..., *θ*_21 _and plot the results in the final column of Figure 2 under the heading "BPSA". Figure 2 shows that stratifying on  does not yield the same degree of balance compared to stratifying on . The point estimates  are generally closer to zero compared to the crude analysis, indicating that some of the association between *X *and *C *has been reduced. However, BPSA does not succeed in balancing the covariates as effectively as PSA. Therefore, BPSA appears to be less effective for controlling confounding than PSA.

The difficulty with this investigation is that it ignores associations between the co-variates and outcome variable. Figure 2 shows that adjusting for  breaks the association between *C*_1_,..., *C*_21 _and *X*. But it does not reveal if these variables are all equally important mortality risk factors. Recall that a covariate *C*_*k *_is defined as a confounding variable if 1) *X **C*_*k*_, 2) *Y **C*_*k*_|*X*, and further that 3) *C*_*k *_is not affected by *X *or *Y *[16]. It is useful to consider the relationship between the covariates and mortality if we wish to identify confounding bias. If certain components of *C *are more strongly associated with *Y *than others, then imbalances in Figure 2 may be misleading.

All of the covariates in the heart failure dataset are a priori known mortality risk factors [17]. But at issue is whether or not they are associated with mortality conditional on the estimated propensity score. The purpose of propensity techniques is to stratify the population into coarse subgroups within which treatment effect estimates are uncon-founded. To get a clear picture of the performance of  and  as tools to control confounding, we should explore the associations between *C *and *Y *conditional on the estimated propensity scores.

## Balance with respect to the outcome

### Review: Prognostic scores for control of confounding

Suppose that  denotes a scalar function of *C*. We say that there is *balance with respect to the outcome *if *Y *╨ *C*|, *X *= 0. This conditional independence assumption says that, among untreated subjects with *X *= 0, the distribution of the outcome is determined by  and does not depend on *C*. Conceptually, the quantity  can be interpreted as a *prognostic score *in the sense that it is a scalar summary of the contribution of *C *to the outcome risk [18].

Hansen [18] recently introduced the notion of prognostic scores for control of confounding in observational studies. For the heart failure data, let *Y*_1 _and *Y*_0 _denote potential outcomes for death for a patient in the study. The quantity *Y*_1 _models mortality at the end of follow up for treated patients and takes value one if the patient dies and zero otherwise. The quantity *Y*_0 _models the corresponding potential outcome for death assuming the patient is untreated. Let *Y *= *Y*_*X *_denote the observed potential outcome.

A prognostic score  is defined as any scalar function of *C *with the property that

(4)

This equation says that  determines the distribution of the outcome among untreated subjects. See Hansen [18] for details.

Prognostic scores are analogous to propensity scores, and they have close connections to disease risk scores reviewed by Rosenbaum and Rubin [1], and Stürmer et al. [19]. Stratifying on a prognostic score removes confounding because it breaks the association between *C *and the outcome. Hansen [18] proves that when a prognostic score  is known, then this implies that we can control confounding by including it as a covariate in a regression model for the outcome. Effect measures calculated from *P*(*Y |X*, ) have a causal interpretation.

The idea of prognostic scores give a theoretical basis for checking balance with respect to the outcome in the heart failure data. To compare the performance of  and  for control of confounding, we can instead verify there is *balance with respect to the outcome*, meaning that *Y *╨ *C*|, *X *= 0. If we see that there is balance with respect to the outcome, what this means is that the estimated propensity score  breaks the association between the covariates and the outcome. Thus by stratifying on , the covariates *C *cease to be outcome risk factors and are therefore no longer confounders.

A crucial feature of prognostic scores is that they do not require that *X *╨ *C*|. If *X **C*|, then effect measures computed from *P*(*Y*|*X*, ) will nonetheless have a causal interpretation provided that equation (4) holds. In other words, just because a covariate summary score does not yield balance with respect to treatment does not imply that it cannot be used to control confounding. We may instead have *Y *╨ *C*|, *X *= 0 in which case  breaks the association between the confounders and outcome.

### Balance with respect to mortality in the heart failure data

To study the ability of  and  to induce balance with respect to the outcome in the heart failure data, we employ a similar empirical investigation strategy to the one described above. We fit models of the form

(5)

where *C*_*k *_denotes the *k*^*th *^component of *C*, the parameter *φ*_*k *_is a regression intercept, and  is a propensity score estimate. Equation (5) is identical to the outcome regression model of equation (1) with *X *= 0, except that we now include the additional covariate *C*_*k *_in the model. It assesses whether or not we should include the covariates *C *in the model in addition to the estimated propensity score. If the estimated propensity score  induces balance with respect to the outcome, meaning that *Y *╨ *C*_*k*_|, *X *= 0, then we should have *ρ*_1 _= *ρ*_2 _= ... = *ρ*_21 _= 0. We may once again compute point estimates  and study the extent to which they depart from zero.

**Figure 3:**
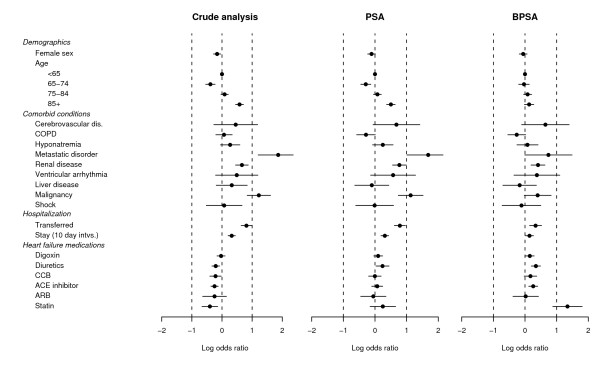
**Balance with respect to the outcome**. Each row corresponds to the log odds ratio (95% CI) for the association between a covariate and mortality, within treatment groups, in either an unadjusted analysis, or after having adjusted for  or .

First we calculate the crude associations between *Y *and *C*_1_,..., *C*_21 _among untreated subjects. This is accomplished by fitting the *p *= 21 regressions in equation (5) while forcing *ω*_*jk *_= 0. In order words, we regress *Y *on each of the individual components of *C*. The results in the form of point and 95% interval estimates of *ρ*_1_,..., *ρ*_21 _are given in the first column of Figure 3 under the heading "Crude analysis". We see that many covariates are strong risk factors for mortality. For example, most of the comorbid conditions are associated with increased risk of death.

Next we examine the performance of  and  as tools to reduce confounding. We fit the 21 regressions in equation (5) substituting either  or  in place of , and computing the corresponding inferences for *ρ*_1_,..., *ρ*_21_. The results are given in the second and third columns of Figure 3 under the headings "PSA" and "BPSA" respectively.

Figure 3 indicates that BPSA produces greater balance with respect to mortality compared to PSA. The point estimates of *ρ*_1_,..., *ρ*_21 _are shifted towards zero. As it stands, the BPSA model *assumes *that the propensity scores achieve balance with respect to the outcome, and the rightmost column of Figure 3 has diagnostic value in supporting or refuting this assumption. So for the present data we see that the assumption is not bad, but not perfect. For example, consider the variable metastatic disorder. In Figure 3, under the heading "Crude analysis" we see that this variable is the strongest predictor of mortality in the heart failure dataset with an estimated log odds ratio of greater than 2. Stratifying on  breaks much of this association, while stratifying on  does not.

To give a clearer comparison of the tradeoffs between BPSA and PSA, Table 2 gives summary statistics for the distribution of the log odds ratios from Figures 2 and 3. For PSA, we see the method gives good balance with respect to treatment because the  are all close to zero. By comparision, BPSA does a better job of reducing the magnitude of the associations between the confounders and outcome that are depicted in Figure 3. For BPSA the sample mean and median are lower compared to PSA. The associations between *C *and *Y *are weaker overall after adjusting for . Similarly, the variance  is 0.12 for BPSA versus 0.24 for PSA. Table 2 does not point decisively towards the superiority of either method. Instead it describes the merits and tradeoffs of the Bayesian approach of using the outcome variable to estimate the propensity scores.

**Table 2:** Summary statistics for the distribution of log odds ratios depicted in Figure 2 and Figure 3.

	**Log odds ratios**			
	**Median**	**IQR**	**Mean**	**Variance**
Balance with respect to treatment				
Crude	0.09	0.68	0.09	0.34
PSA	0.02	0.04	0.02	0.02
BPSA	0.20	0.39	0.19	0.12
Balance with respect to outcome				
Crude	0.08	0.70	0.25	0.31
PSA	0.24	0.58	0.31	0.24
BPSA	0.15	0.35	0.23	0.12

## Conclusion

In the population of heart failure patients, confounding from *C *is driven by associations between *C *and *Y*, as well as by associations between *C *and *X*. By conditioning on , the Bayesian propensity score method yields strata where we have roughly *Y *╨ *C*|*X*, *Z*. Confounding is reduced because *C *are no longer strong mortality risk factors. By fitting regressions for *X *and *Y *simultaneously, BPSA treats the propensity score as a predictor for the outcome. The tradeoff is that BPSA is less successful in balancing the confounders with respect to the treatment variable.

In contrast, PSA estimates propensity scores from the marginal model for *X *given *C*. The method handles all components of *C *similarly, regardless of the strength of their association with *Y*. Figure 2 reveals that PSA balances all covariates equally well, but the approach may be overly pessimistic. Some covariates are more important mortality risk factors than others. It should be emphasized that neither method is able to reduce confounding from unobserved covariates.

A limitation of BPSA is that the propensity score estimates cannot be used to study multiple outcomes. Whereas traditional propensity scores ignore the outcome variable, Bayesian propensity scores are outcome specific. Equation (1) makes use of modelling assumptions for the relationship between *Z *and the particular outcome *Y *under investigation. Indeed the strategy of fitting models for the treatment and outcome simultaneously goes against the idea of setting up the study design and analysis without access to the outcome [20]. Nonetheless, some authors argue that the performance of propensity score techniques is improved by making use of the outcome data. If our objective is to control confounding, then variables that are weakly associated with the outcome are less important in propensity score modelling, regardless of whether they are strong predictors of treatment [6,12,20,21].

From a substantive point of view, one can argue that equation (1) is not realistic in the sense that we do not expect to have *Y *╨ *C*|*X*, *Z*. Nonetheless, in regression adjustment for the propensity score, the investigator must choose a model for the relationship between the outcome and the propensity score. Popular choices include stratifying on subclasses of the propensity score or assuming a linear relationship. The model should be based on genuine beliefs about the relationship between the propensity score and the outcome. Further discussion of model based regression adjustment for the propensity score is given by Rosenbaum and Rubin [1].

## Competing interests

The authors declare that they have no competing interests.

## Authors' contributions

LM conducted the analysis, interpretation of results, and drafting and revising the manuscript. PG and PA contribute to conceiving and interpreting the analysis as well as drafting and revising the manuscript. AL contributed to acquisition of the data and revising the manuscript. All authors read and approved the final manuscript.

## Supplementary Material

Additional file 1 **Code for fitting Bayesian propensity analysis to a toy synthetic dataset**. Computer code for implementing BPSA in the software package R. Click here for file
